# Fully-automated production of [^68^Ga]Ga-PentixaFor on the module Modular Lab-PharmTracer

**DOI:** 10.1186/s41181-020-0091-2

**Published:** 2020-02-27

**Authors:** Sarah Spreckelmeyer, Oliver Schulze, Winfried Brenner

**Affiliations:** Charité - Universitätsmedizin Berlin, corporate member of Freie Universität Berlin, Humboldt-Universität zu Berlin, and Berlin Institute of Health, Department of Nuclear Medicine, Augustenburger Platz 1, 13353 Berlin, Germany

**Keywords:** Automated production, Imaging, CXCR4, PentixaFor

## Abstract

**Background:**

PentixaFor is a promising radiopharmaceutical for positron emission tomography in the detection of different tumor entities and other diseases. Until now, the synthesis of [^68^Ga]Ga-PentixaFor was reported for the automated synthesis module from Scintomics® only. Our aim was to evaluate the automated synthesis of this radiopharmaceutical on a different module in order to make it available for a broader community.

**Results:**

The synthesis of [^68^Ga]Ga-PentixaFor with different amounts of PentixaFor (50 μg, 30 μg and 20 μg) on the Modular Lab PharmTracer (MLPT) from Eckert & Ziegler with the already established synthesis template for [^68^Ga]Ga-DOTATOC yielded best results with 50 μg PentixaFor for clinical multi-dose application. All different quality control parameters tested (e.g. sterility, stability and radiochemical purity) were in accordance with the European Pharmacopoeia.

**Conclusions:**

[^68^Ga]Ga-PentixaFor was successfully synthesized fully-automated on the synthesis module Modular Lab PharmTracer and can be used for multi-dose application in clinical settings.

## Background

**Fig. 1:**
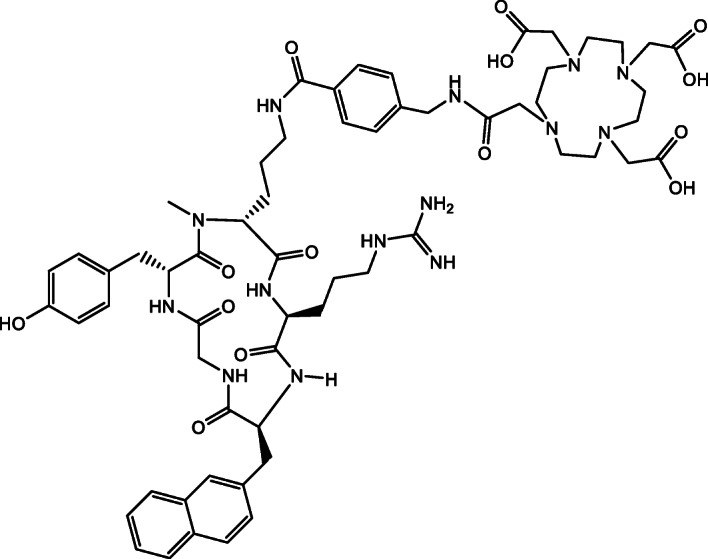
Structure of PentixaFor (molecular weight: 1221.4 g/mol)

In 2011, Demmer et al. first introduced chemokine receptor-4 (CXCR4)-binding peptidic probes for molecular imaging (Demmer et al., 2011). PentixaFor (Synonym for CPCR4.2, Fig. 1) is to date the most promising peptide with a high affinity to chemokine receptor-4. PentixaFor can be used as PET imaging agent when coupled to the positron emitting radionuclide gallium-68. Numerous clinical studies have already been performed in vivo and the interest is steadily increasing in new applications for different tumor entities and other diseases, e.g. inflammatory conditions (Bouter et al., 2018). Examples of clinical applications in oncology are the detection of neuroendocrine tumors, multiple myeloma (Pan et al., 2018), glioma, leukemia (Mayerhoefer et al., 2018) or lymphoma (Luo et al., 2019; Haug et al., 2019). Since the precursor PentixaFor was made available until summer 2018 only for use by the synthesis module from Scintomics®, only hospitals that operated a Scintomics® module were able to prepare [^68^Ga]Ga-PentixaFor. Here, we would like to introduce the synthesis conditions of [^68^Ga]Ga-PentixaFor on a different synthesis module, namely Modular Lab PharmTracer (MLPT) from Eckert & Ziegler Eurotope GmbH – in order to introduce [^68^Ga]Ga-PentixaFor to a broader community. For that purpose, different concentrations of PentixaFor were evaluated based on quality control parameters. Concerning the synthesis of ^68^Ga-tracers in general, different modules are available on the market which have been reviewed by Boschi et al. In addition, generator post-processing steps like fractionation, anionic-exchange and cation-exchange are described (Boschi et al., 2013). The now proposed fully automated pre-purification of gallium-68 on a strong cation exchange (SCX) cartridge has the advantage that gallium-68 becomes concentrated and germanium-68 as well as non-radioactive impurities (e.g. Zn^2+^) are trapped on the SCX cartridge.

## Results

### Labeling results of different amounts of PentixaFor

**Fig. 2:**
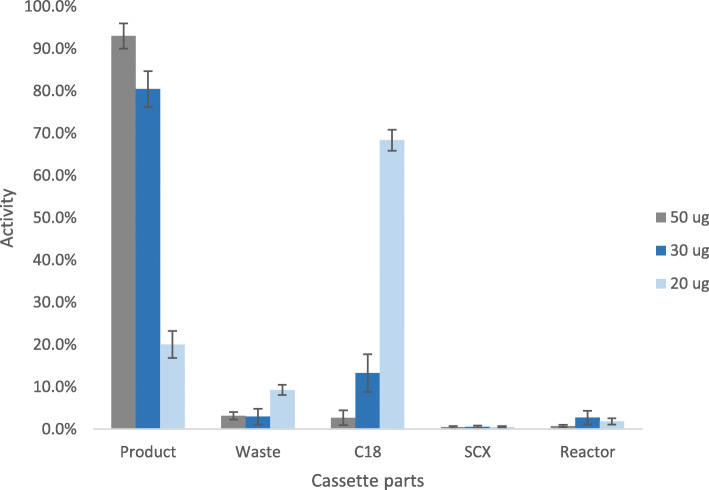
Overview activity distribution [% of total] of different experiments n ≥ 3 (grey: 50 μg; blue: 30 μg; light blue: 20 μg)

The fully automated production of [^68^Ga]Ga-PentixaFor was conducted on the commercial labeling synthesis module MLPT. Three different precursor amounts of PentixaFor were tested for radiolabeling with 1080–2500 MBq of gallium-68 (three generators from type GalliaPharm®) - 50 μg, 30 μg and 20 μg. As shown in Table 1, the highest amount of PentixaFor (50 μg) resulted in 94.7% radiochemical yield (RCY, n.d.c.) in the product vial, 2.6% in the waste vial and 1.7% on the C_18_ cartridge. By lowering the amount of PentixaFor to 30 μg, the RCY of the product decreased to 80.5% (n.d.c.) and the amount on the C_18_ cartridge increased to 13.3% (n.d.c.). By lowering the amount of starting material to 20 μg, the efficiency became worse. Only 20.0% (n.d.c.) of radiochemical yield was found in the product vial and 69.4% on the C_18_ cartridge. The distribution of the radioactivity in the cassette for the productions is presented in Fig. 2.

**Table 1:** Activity measured on different parts of the cassette immediately after synthesis (*n* ≥ 3)

	Product n.d.c. [%]	Waste n.d.c. [%]	C_18_ cartridge n.d.c. [%]	RCY (decay corrected) [%]
50 μg	94.7 ± 0.7	2.6 ± 0.5	1.7 ± 0.5	80.9 ± 10.0
30 μg	80.5 ± 4.2	2.9 ± 1.8	13.3 ± 4.5	71.0 ± 10.6
20 μg	20.0 ± 3.2	9.3 ± 1.2	68.4 ± 2.5	18.9 ± 2.4

The radioactivity found in the reactor and SCX cartridge did not change significantly by changing the amount of starting material.

In the waste fraction, with decreasing peptide amounts from 50 to 20 μg, the radioactivity increased from 2.6% to 9.3%. Interestingly, the activity measured in the waste vial consists of approximately 25% product and 75% free gallium-68 as seen in the radio-HPLC chromatogram as well as in the radio-iTLC (Supporting Information, Fig. 1).

### Quality control of [^68^Ga]Ga-PentixaFor

The radiochemical purity was evaluated with a standard protocol by radio-HPLC and radio-iTLC.

**Fig. 3:**
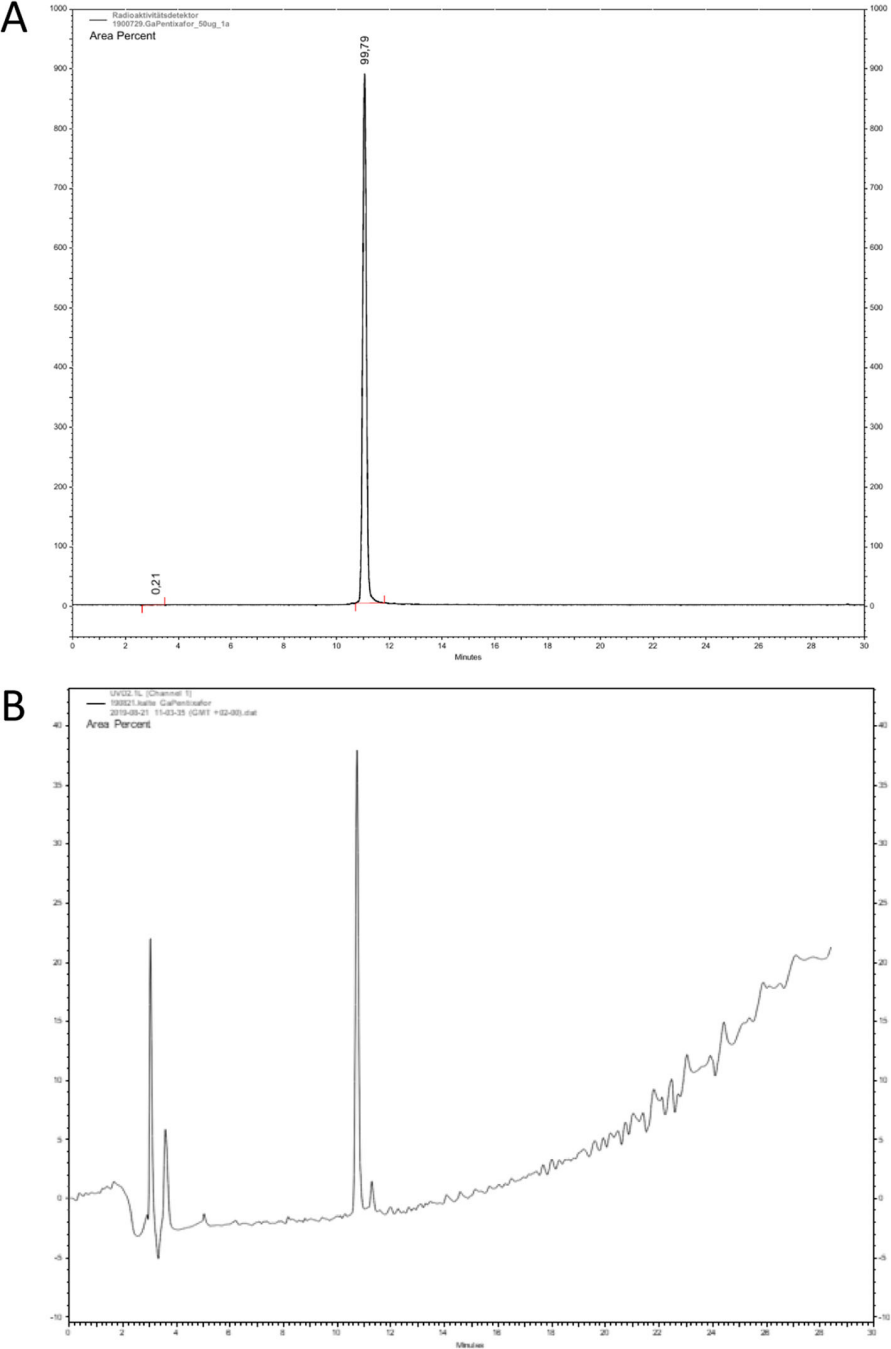
**a** Radio-HPLC Chromatogram of [^68^Ga]Ga-PentixaFor, 50 μg. **b** UV-HPLC Chromatogram of [^nat^Ga]Ga-PentixaFor

With radio-HPLC, free gallium-68 would be detected at t_R_ = 2.5 min, whereas gallium-68 bound to PentixaFor was detected at t_R_ = 11 min. Radioactive impurities could not be detected with this method (Fig. 3a).

**Fig. 4:**
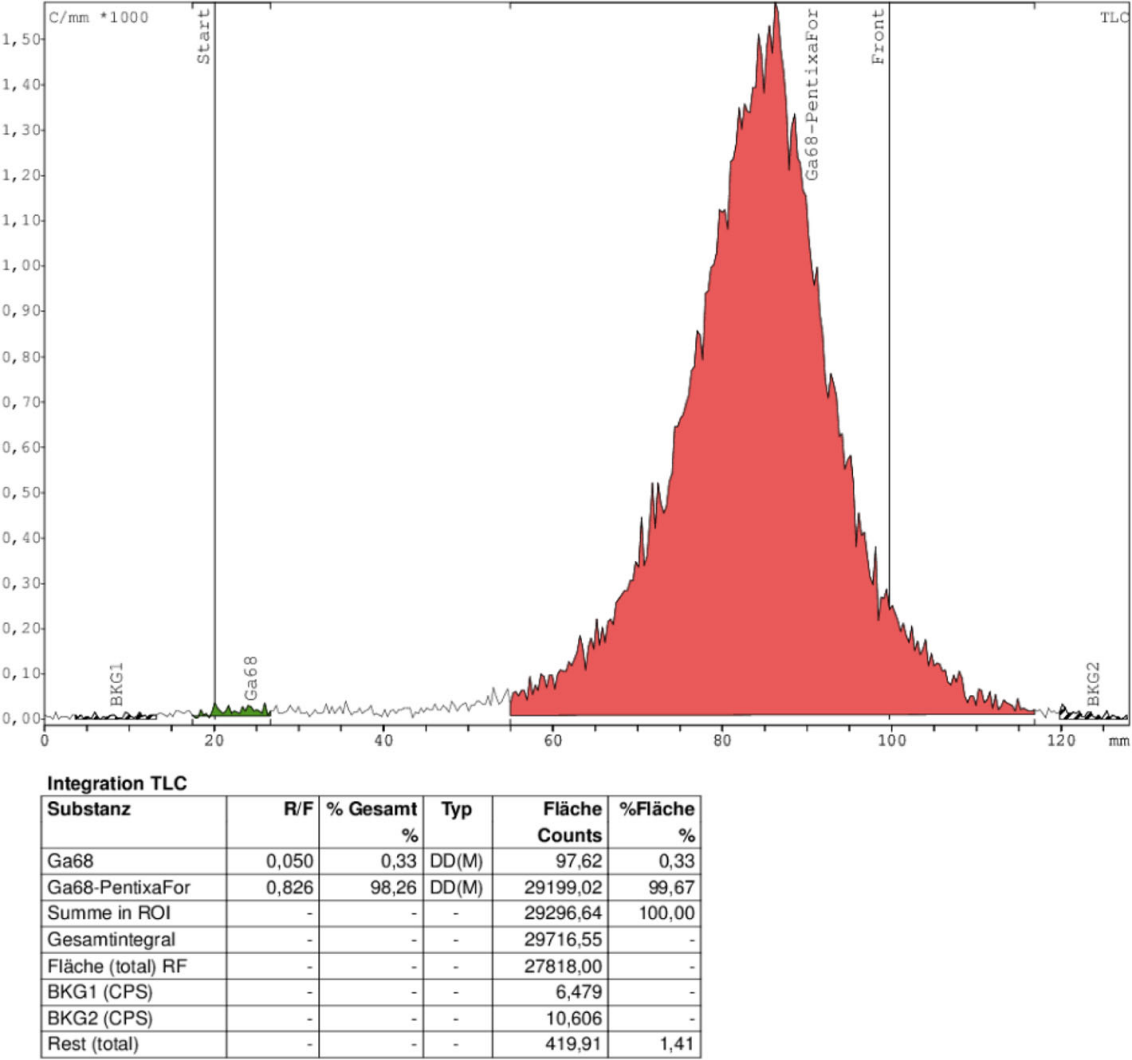
iTLC- Chromatogram of [^68^Ga]Ga-PentixaFor, 50 μg

With radio-iTLC, no ^68^Ga-colloide could be detected at R_f_ = 0.2 but the product at R_f_ = 0.8 (Fig. 4).

The cold standard [^nat^Ga]Ga-PentixaFor shows a similar retention time of t_R_ = 11 min as seen in Fig. 3b.

In addition, the product solution was tested for endotoxins. For this purpose, the solution was diluted with endotoxin-free-water in a ratio 1:10. For all samples, an endotoxin concentration below 0.5 IE/mL was detected. This is in accordance with the European Pharmacopoeia (9.0/0125).

With regards to sterility, all products were sterile.

**Fig. 5:**
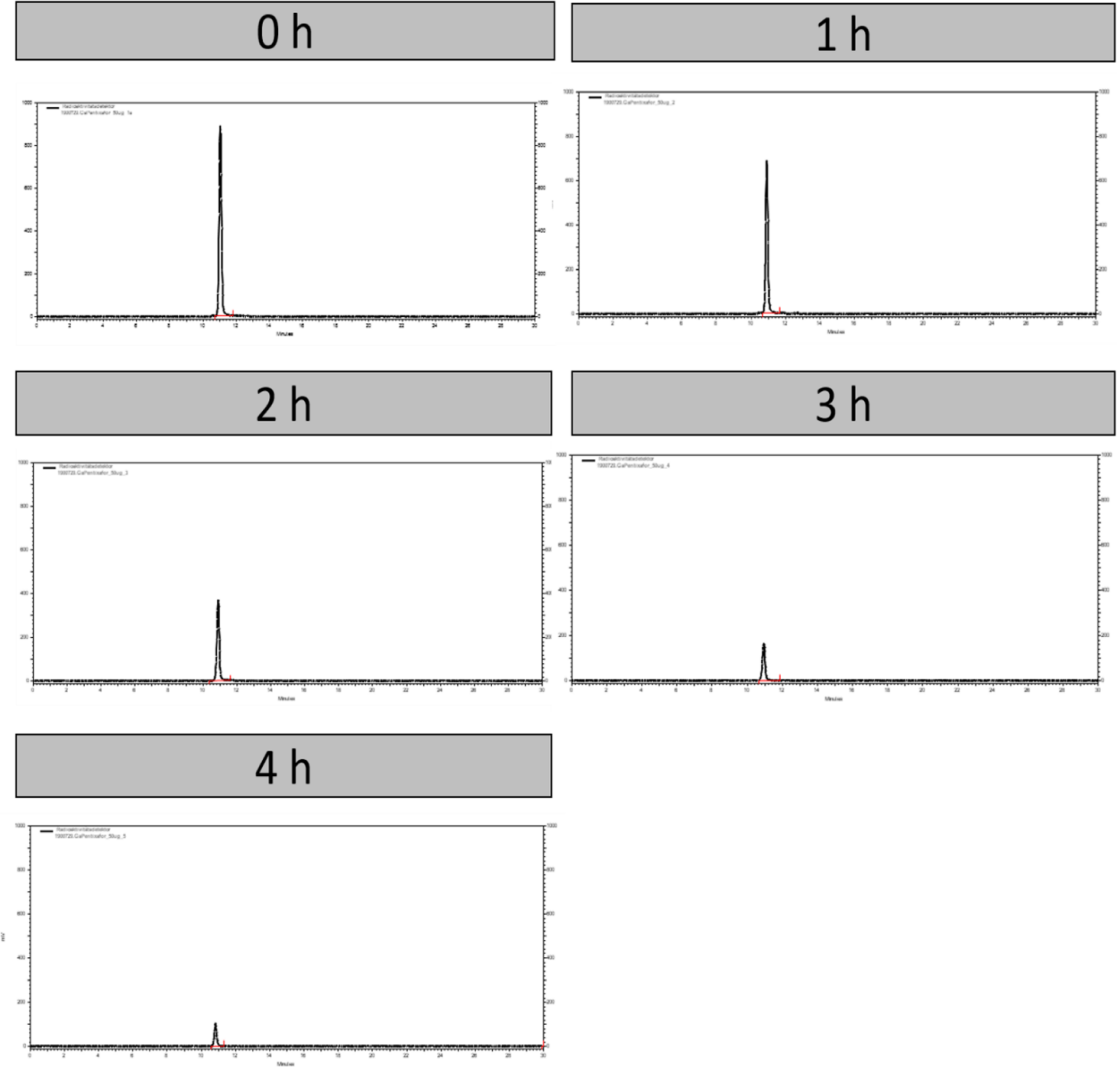
Stability of 50 μg [^68^Ga]Ga-Pentixafor

The stability of [^68^Ga]Ga-PentixaFor in aqueous solution at room temperature was tested up to 4 h via radio-HPLC. As seen in Fig. 5, [^68^Ga]Ga-PentixaFor is stable at those conditions. No radioactive by-products or free gallium-68 could be detected during this time period.

In Table 2, product specifications of the most promising approach with 50 μg PentixaFor are summarized. Acceptance criteria are based on the European Pharmacopoeia (9.0/0125).

**Table 2:** Summary of the product specifications for 50 μg PentixaFor, n ≥ 3

Test	Acceptance criteria	[^68^Ga]Ga-PentixaFor
Radiochemical purity (radio-HPLC)	> 95%	99.8%
Radiochemical purity (radio-iTLC)	> 95%	99.7%
pH	4–8.5	5
Radioactivity concentration	> 50 MBq/ mL	120–190 MBq/ mL
Radioactivity	> 150 MBq	1080–1700 MBq (depending on amount of generators used)
Volume	2–10 mL	9 mL
Color	Colorless	Colorless
Molar radioactivity	1-60 MBq/ nmol	27-42.5 MBq/nmol
Radionuclidic purity	> 99.9%	99.9 %
^68^Ge breakthrough	< 0.001%	0.0002%
Endotoxins	< 19.0 IE/mL	< 0.5 IE/mL
Stability over 4 h	> 90%	99.9%
EtOH content	< 10%	≤ 6.2% (calculated)

## Discussion

For establishing the automated synthesis of [^68^Ga]Ga-PentixaFor on the Modular Lab PharmTracer from Eckert & Ziegler Eurotope GmbH for the first time, three different amounts of PentixaFor were evaluated for the production of this tracer with the same standard synthesis template as used for [^68^Ga]Ga-DOTATOC on the synthesis module MLPT. The results summarized in Table 1 and Fig. 2 demonstrate that the amount of 50 μg of PentixaFor yields the best radioactivity distribution on the cassette. The low standard deviations for 50 μg confirm the high reproducibility of the production.

For 30 μg and 20 μg, an increasing amount of radioactivity on the C_18_ cartridge and a decreasing yield of activity in the product vial was observed. Theoretically, two scenarios are to our knowledge possible to explain the increased radiochemical yield on the C_18_ cartridge. Either the product is trapped on the C_18_ cartridge or uncomplexed ^68^Ga-colloide. Product could be eluted from the C_18_ cartridge with pure ethanol. After the automated synthesis is completed, we tried to elute the activity. This resulted in no change of activity measured on the C_18_ cartridge. As a consequence, no product is stuck on the C_18_ cartridge. The most likely explanation is that 20 μg and 30 μg are too low to complex ^68^Ga^3+^ ions completely resulting in a trapping of unbound gallium-68 in the form of ^68^Ga^3+^-colloide on the C_18_ cartridge.

Based on the analyzation of the waste fraction by radio-HPLC and radio-iTLC, 25% of the activity found in the waste fraction was product. With respect to the total radioactivity used in 50 μg experiments, this means a negligibly product activity loss of under 1%.

The minimum amount needed to fully complex PentixaFor under the described conditions was 50 μg. In former studies in which [^68^Ga]Ga-PentixaFor was synthesized on a synthesis module from Scintomics®, 40 μg and 20 μg of PentixaFor were used in a final product volume of 15 mL and 14 mL, respectively. The amount of PentixaFor that is safe to be used in vivo is therefore < 20 μg per patient based on those results (Lapa et al., 2016; Wester et al., 2015). In the frame of the here described synthesis protocol, it is important to make sure via standard operating procedures (SOPs), that the maximal injected amount of tracer is below 20 μg/patient. Thus, the maximum injected volume needs to be below 3.6 mL.

With regard to the quality control parameters, we achieved an endotoxine-free, sterile and stable solution of [^68^Ga]Ga-PentixaFor with a radiochemical purity > 95% over a period of 4 h. Moreover, all tested quality control parameters were in accordance with the European Pharmacopoeia (see Table 2). Calculating the ethanol content based on the parameters chosen in the synthesis template of the MPLT software, results in an ethanol content that cannot exceed 6.2%. Nevertheless, we recommend to either measure the ethanol content of every batch by gas chromatography before releasing it for clinical application, or validate the ethanol content by an external laboratory if a gas chromatography is not available onsite.

## Conclusions

[^68^Ga]Ga-PentixaFor was successfully synthesized fully-automated on the synthesis module MLPT for the first time. All the tested quality parameters for the radiochemical purity, pH, endotoxins and sterility are in accordance with the European Pharmacopoeia. In addition, the product solution is stable for at least 4 h after production, as shown by radio-HPLC. Thus, [^68^Ga]Ga-PentixaFor can be easily and reliably produced on the module MLPT for clinical application.

## Methods

### Materials

PentixaFor was obtained from PentixaPharm. An aqueous stock solution of 1 mg/mL was prepared and kept at − 15 °C. All chemicals were of pure chemical grade and solvents for high-pressure-liquid-chromatography (HPLC) were obtained as HPLC grade. TraceSelect water (Sigma-Aldrich) was used in all experiments. The pharmaceutical grade ^68^Ge/^68^Ga generator (GalliaPharm®, Eckert & Ziegler Radiopharma GmbH, Germany), Modular Lab PharmTracer (Eckert & Ziegler Eurotope GmbH, Germany) and reagent set EZ-102 (Eckert & Ziegler Eurotope GmbH, Germany) were used. The amount of detected metal impurities/ ^68^Ge breakthrough as provided by the manufacturer was less than the defined limit in the European Pharmacopeia monograph. Activity counting was determined using a borehole counter (Nuklear-Medizintechnik Dresden GmbH, Germany). HPLC was performed using the HPLC system Knauer Azura (UVD: 2.1 L; P6.1L) coupled with UV and radiometric (Raytest Socket 2″8103 0370) detectors. The TLC scanner used was MiniGita from Raytest. The test for endotoxins was performed using Nexgen PTS (Charles River).

### Preparation for labeling of PentixaFor with gallium-68

First, the commercial fully automated synthesis platform MLPT was equipped with a disposable single-use cassette (C4-GA-PEP). The synthesis template and buffer preparations were identical to the ones used for [^68^Ga]Ga-DOTATOC. All synthesis reagents were contained in the reagent set except the peptide. From the reagent set, 50 mL of 0.9% NaCl and 10 mL of EtOH/H_2_O (50/50) solution were connected to the cassette on the designated spikes. After connection of the waste vial the preconditioning of the C_18_ cartridge was performed automatically without user interaction. During the conditioning step PentixaFor was prepared in 20 μg, 30 μg and 50 μg aliquots from the stock solution (1 mg / mL). Meanwhile, the preparation of the acetate buffer solution was performed according to the user manual using EZ 102. The reaction mixture contains 2.2 mL of the final buffer solution, 0.4 mL of EtOH/H_2_O (50/50) and an aliquot of PentixaFor, which was loaded into the reaction vial. The required volume of eluent was added to the eluent vial prior to synthesis.

### Labeling of PentixaFor with ^68^GaCl_3_

The synthesis was performed fully-automated without any user interaction. ^68^Ga^3+^ obtained from a 1.850 MBq ^68^Ge/^68^Ga generator (GalliaPharm®) with TiO_2_ matrix, was eluted with 0.1 N HCl. The generator eluate was pre-concentrated on a strong cation exchange (SCX) cartridge. [^68^GaCl_4_]^−^ was recovered from the SCX cartridge by the eluent (5 M NaCl/HCl (0.1 M)) (Schultz et al., 2013). The reaction vial containing the reaction mixture was preheated to 50 °C, and after the ^68^Ga-activity was transferred to the reaction vial, the temperature was increased to 95 °C. After 5 min at 95 °C, the reaction mixture was cooled down by adding 3 mL of 0.9% NaCl. The crude reaction solution was subsequently transferred to the C_18_ cartridge for purification. The final product was eluted from the C_18_ cartridge using a EtOH/H_2_O (50/50) solution and passed through a 0.22 μm sterile filter into the sterile product vial. The resulting product was diluted to 9 mL with 0.9% NaCl. A sample for quality control was taken.

### Quality control

After synthesis, the product vial is removed from the MLPT module and further evaluated for quality control determining the following parameters: total product activity, ^68^Ga^3+^-identity via half-life time, chemical purity (pH, sterility and endotoxins) and radiochemical purity (high-pressure-liquid chromatography). The stability of the product at room temperature was monitored by radio-HPLC for 4 h. Radio-HPLC was performed with a standardized method. A: water + 0.1% TFA; B: acetonitrile + 0.1% TFA, gradient: 0–25 min A: 0–100%; 25–28 min A: 100–0%. The limit of detection of the radio-HPLC is 10 kBq/ 20 μL injected volume and the recovery is approximately 80%. For iTLC, ammonium acetate/methanol (1/1) was used as mobile phase and iTLC-SG strips as stationary phase. The sterility tests were performed as described in the European Pharmacopoeia at the Institut für Hygiene, Charité.

## Supplementary information

**Additional file 1. Supporting Information, Fig. 1:** iTLC chromatogram of the waste fraction

## Data Availability

All data generated or analyzed during this study are included in this published article.

## Abbreviations

^68^Ga^3+^: ^68^Gallium^3+^ ion
^68^Ge^4+^: ^68^Germanium^4+^ ion
CXCR4: Chemokine receptor-4
HPLC: High-pressure-liquid-chromatography
MLPT: Modular Lab PharmTracer
PET: Positron Emission Tomography
RCY: Radiochemical yield
TFA: Trifluoro acetic acid
