# Supplementary figures and images for: Fully-automated production of [68Ga]Ga-PentixaFor on the module Modular Lab-PharmTracer
Source: EJNMMI Radiopharm Chem. 2020 Feb 27;5:8. doi: 10.1186/s41181-020-0091-2 (PMC7046903; doi:10.1186/s41181-020-0091-2)

## Slide 1
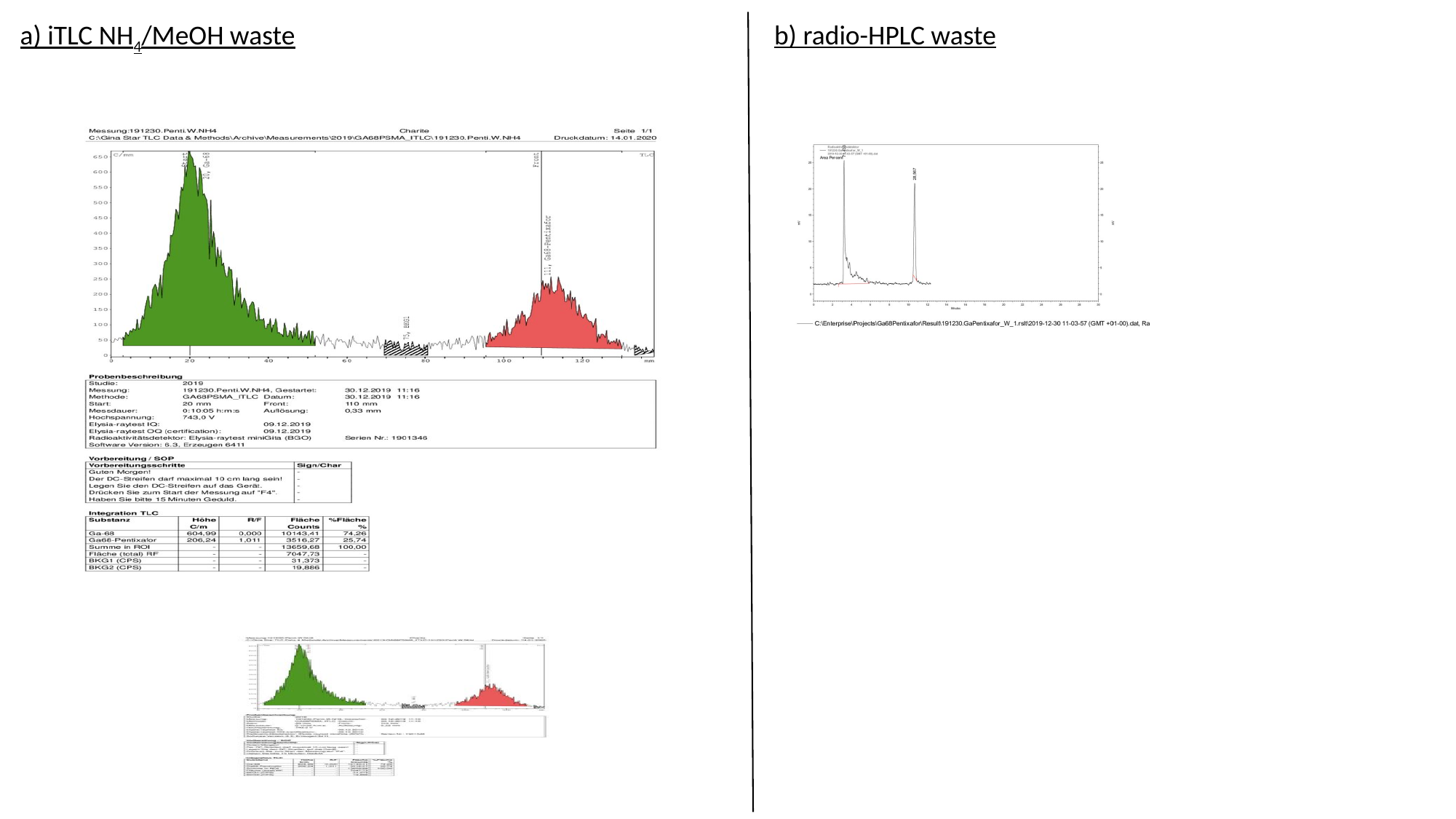

b) radio-HPLC waste
a) iTLC NH4/MeOH waste

Supplement: Supplementary file 1 — Additional file 1. Supporting Information, Fig. 1: iTLC chromatogram of the waste fraction [file 41181_2020_91_MOESM1_ESM.pptx]
